# An Improved Genetic Fuzzy Logic Control Method to Reduce the Enlargement of Coal Floor Deformation in Shearer Memory Cutting Process

**DOI:** 10.1155/2016/3973627

**Published:** 2016-04-27

**Authors:** Chao Tan, Rongxin Xu, Zhongbin Wang, Lei Si, Xinhua Liu

**Affiliations:** ^1^School of Mechatronic Engineering, China University of Mining & Technology, Xuzhou 221116, China; ^2^Xuyi Mine Equipment and Materials R&D Center, China University of Mining & Technology, Huai'an, China; ^3^School of Information and Electrical Engineering, China University of Mining & Technology, Xuzhou 221116, China

## Abstract

In order to reduce the enlargement of coal floor deformation and the manual adjustment frequency of rocker arms, an improved approach through integration of improved genetic algorithm and fuzzy logic control (GFLC) method is proposed. The enlargement of coal floor deformation is analyzed and a model is built. Then, the framework of proposed approach is built. Moreover, the constituents of GA such as tangent function roulette wheel selection (Tan-RWS) selection, uniform crossover, and nonuniform mutation are employed to enhance the performance of GFLC. Finally, two simulation examples and an industrial application example are carried out and the results indicate that the proposed method is feasible and efficient.

## 1. Introduction

With the development of coal mining technology and stringent requirement for colliery safety, the automation of fully mechanized coal face has been inevitable trend. As a major coal mining machine, shearer plays a pivotal role in getting high-security and high efficiency of exploitation [1, 2]. In order to realize the automated control of shearer, multiple coal-rock interface recognizing and tracking methods [3–6] were proposed, but these methods are not satisfactory because of the poor working conditions of coal mining such as narrow space, high coal dust, low visibility, and large noise [7]. Memory cutting as a better indirect solving strategy than aforementioned methods has been emphasized [8].

In recent years, many scholars have been dedicated to researching and developing the shearer memory cutting methods. Related studies mainly focus on the automatic height adjusting method of shearer front drum and the adjustment of shearer drawing speed: Li et al. adopted grey-Markovian memory cutting algorithm to improve the efficiency and accuracy of shearer height adjusting [9]; Li et al. proposed a hidden Markov model (HMM) method for shearer memory cutting to solve the problem of large residual errors and frequent adjustments of drums [10]; Su et al. combined sliding-mode variable structure control strategy with shearer memory cutting to make the automatic height adjusting stable and fast [11]; Wang et al. carried out the self-adaptive shearer memory cutting based on artificial immune theory and the automatic adjustment height of the drum was realized [12]; Xu and Wang built a shearer self-adaptive memory cutting model based on fuzzy control theory to adjust the drawing speed and drum height self-adaptively [13]. Few studies notice the bad influences caused by poor coal floor conditions. Zhou et al. are the first to expound the problem of shearer memory cutting caused by variant coal floor and proposed a novel approach based on the fuzzy algorithm to improve the implementation precision of shear memory cutting [14]. However, the abovementioned studies have common disadvantages. Firstly, most of the proposed approaches are applied only considering front drum memory cutting without considering back drum memory cutting. Secondly, no literature notices that the coal floor deformation and its enlargement negatively affect the regular work of shearer. The shape of initial coal floor is usually concavo-convex, which brings up the concavo-convex walking path in following implementation process. Finally, the coal floor deformation will get enlarged. This phenomenon cannot guarantee the regular work of shearer and the shearer operators need to adjust the rocker arms manually and frequently to avoid drums cutting rocks. Moreover, excessive deformations may cause drum damage and then lead to security accident. Under this kind of background, it is necessary to study the strategy for reducing the enlargement of coal floor deformation.

In actual coal mining field, the underground conditions are complicated and unstable so that the universal mathematic model is hard to elaborate. To solve this kind of problem, fuzzy logic control (FLC) method is commonly used. FLC can imitate cognition and experience of human beings to depict the production process [15–17], and it can deal with imprecise information through linguistic expressions. In recent years, FLC has been successfully applied to numerous complicated issues that cannot be described with comprehensive mathematic model. FLC system normally consisted of five parts: fuzzification, rule base, database, inference engine, and defuzzification. Fuzzification, inference engine, and defuzzification are exactly similar in most studies, but the rule base and database are often designed subjectively, which makes the logic rules and the membership functions cannot be adjusted adaptively [18].

In recent years, many researchers employ genetic algorithm (GA) to optimize the rule base and database. As an optimized fuzzy control system, GFLC has been applied to many fields and achieved remarkable effects [19–22]. However, the performance of GA has major impacts on the control effect of GFLC. Standard GA is most frequently faced with many drawbacks, such as premature convergence, local optimal deficiency, and no capacity of adaption [23].

Many researchers have proposed effective improvement strategies and they can be generally divided into 4 categories: (1) improving encoding methods; (2) enhancing the generating methods of initial population; (3) optimizing genetic operation; (4) adopting the adaptive probability of crossover and mutation. Yang et al. proposed several improvement strategies for GA to optimize operation of cascade reservoirs, such as solution space generation, chaos optimization for initial population, new selective operators, and adaptive probabilities of crossover and mutation [23]. Ye et al. proposed a parameter-adjusting method to adaptively adjust crossover rate and mutation rate [24]. He et al. used locus-independent selection strategy for real-coded GA [25]. Misevius and Rubliauskas proposed superindividuals method to accelerate the convergence speed and improve the qualities of individuals [26]. In [27], GA with new coding and operators is employed to optimize capacitor placement. Zhang and Wong proposed an objective-coding GA to resolve integrated process planning and scheduling problems [28].

Nevertheless, most improvement strategies are based on binary encoding and real encoding, using such encoding methods to encode the fuzzy logic rules and membership functions of FLC may result in the over length of chromosome and the low operating speed of GA. The integer encoding reduces the length of chromosome and lowers the complexity of decoding [29]; thus it is more suitable for GFLC.

Bearing the above observation in mind, the GFLC is used to reduce the enlargement of coal floor deformation. To guarantee the performance of GFLC, some GA constituents are employed. Famous uniform crossover and the nonuniform mutation are used to suit the integer encoding; a tangent function roulette wheel selection (Tan-RWS) method is used to improve the GA and speed up convergence; adaptive probability of crossover and mutation is employed in addition. The rest of this paper is organized as follows. The problem description is presented in Section 2. The integrated approach is proposed in Section 3. In Section 4, the proposed approach is proved to be efficient by simulation results based on the data from industrial production scene. An industrial example of mine automation production based on proposed system is demonstrated to specify the application effect in Section 5. Our conclusions and future works are summarized in Section 6.

## 2. The Enlargement Problem of Coal Floor Deformation

The shearer memory cutting consists of two stages: demonstration and implementation [14]. In demonstration stage, the shearer rocker arms are adjusted manually by operators to follow the coal-rock interface. Meanwhile, the shearer gesture data can be collected and saved into the storage unit of shearer controller. In implementation stage, the shearer can repeat the cutting path automatically according to the memory data saved in the controller.

**Figure 1:**
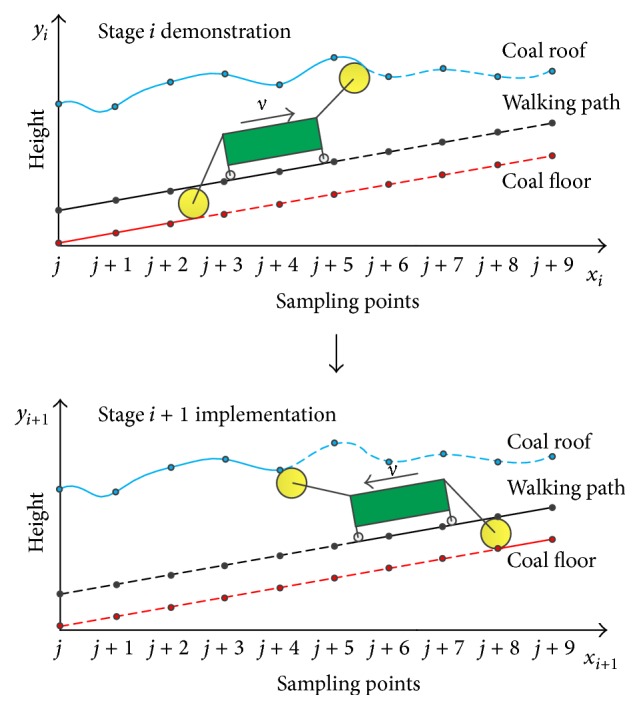
Ideal shearer memory cutting process.

An ideal shearer memory cutting process is shown in Figure 1, and the walking path and coal floor in demonstration stage (stage *i*) are straight lines. After the demonstration stage is completed, the scraper conveyer is pushed onto the coal floor, making the walking path in implementation stage (stage *i* + 1) have the same shape as the coal floor in stage *i*. Then, the implementation stage is carried out according to the memory data. Finally, the coal floor cut in stage *i* + 1 is also straight lines.

**Figure 2:**
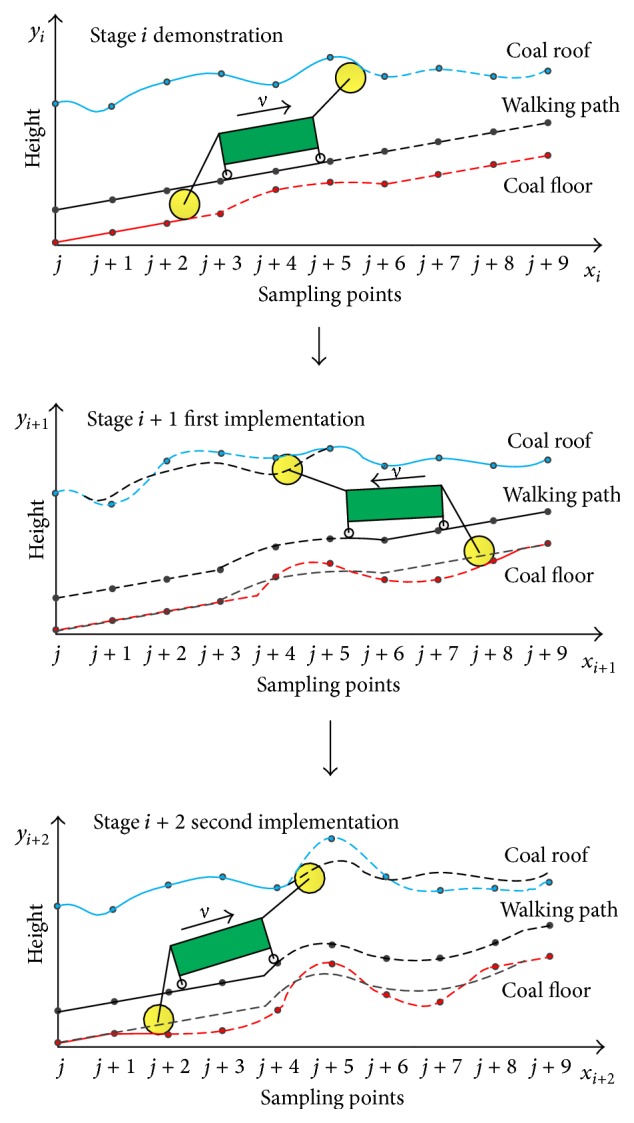
Coal floor deformation and enlargement occurred in the process of shearer memory cutting.

However, deformations such as protuberance and sinking always exist on the coal floor. As shown in Figure 2, a protuberance occurs on the coal floor in stage *i*; thus a protuberance will occur on the walking path in stage *i* + 1 accordingly. If the shearer works according to the memory data, a larger deformation will occur on the coal floor. Moreover, the coal roof is also affected. In stage *i* + 2, the deformation will get even larger. In this paper, this phenomenon can be defined as the enlargement of coal floor deformation. Along with the enlargement, the peak of the coal roof gets higher, and the bottom of the coal floor gets lower. When the deformation is enlarged to a certain extent, the drum may be harmed by the rock.

Through the adjustment of the rocker arm angle in implementation stage, the enlargement of coal floor deformation can be reduced. In order to find the essential factors in the adjustment of rocker arm angle, the shearer is moved into a 2D plane and a simplified model can be built.

**Figure 3:**
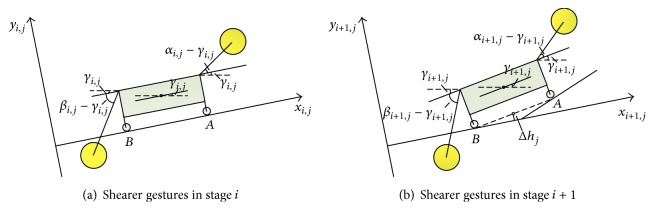
Shearer gestures in stage *i* and stage *i* + 1.

**Figure 4:**
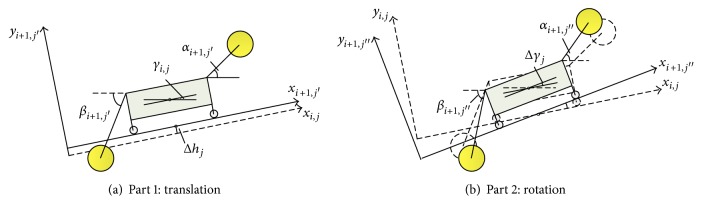
Two parts of stage *i* + 1.

The shearer gestures in stage *i* can be illustrated as Figure 3(a), and *α*_*i*,*j*_, *β*_*i*,*j*_, and *γ*_*i*,*j*_ represent the front rocker arm angle, back rocker arm angle, and fuselage angle, respectively. In stage *i* + 1, deformation occurs on the walking path, and it can be illustrated in Figure 3(b), the center of *AB* is defined as walking center, and Δ*h*_*j*_ is the deviation of the walking center height between stage *i* and stage *i* + 1. In order to facilitate the analysis, stage *i* + 1 can be seen as a combination of two parts: Part 1 and Part 2; Part 1 is the translation of stage *i* by Δ*h*_*j*_ and Part 2 is the rotation of stage *i* by Δ*γ*_*j*_, as shown in Figure 4. From Figure 4, we can find that Δ*h*_*j*_ and Δ*γ*_*j*_ are two essential factors in the process of adjusting rocker arm angle.

## 3. The Proposed Approach

### 3.1. Framework of the Proposed Approach

**Figure 5:**
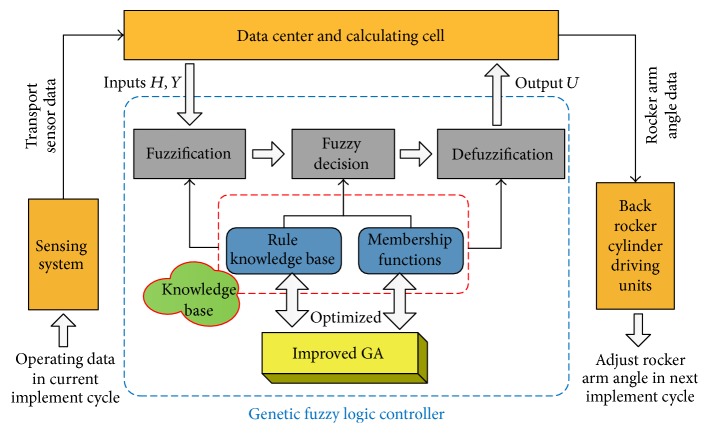
Framework of proposed approach.

In this paper, the deviation of the walking center height between current implementation stage and demonstration stage is marked as *H* and the change of fuselage angle between the two stages is marked as *Y*. Then, *H* and *Y* are taken as the inputs and the adjustment of coal floor height in next implementation stage *U* is the output. The framework of proposed approach can be depicted in Figure 5.

### 3.2. Fuzzification and Encoding

The fuzzy sets corresponding to *H*, *Y*, and *U* are associated with seven linguistic values “NL,” “NM,” “NS,” “Z,” “PS,” “PM,” and “PL,” which represent large negative, medium negative, small negative, zero, small positive, medium positive, and large positive, respectively. According to the linguistic values, the encoding methods for fuzzy logic rules and membership function can be described as follows.

#### 3.2.1. Encoding of Fuzzy Logic Rule

The encoding method proposed by Thrift [29] can effectively shorten the length of chromosome for encoding fuzzy logic rules. The fuzzy logic rules shown in Table 1 are taken as an example. On the basis of the 7 linguistic values of *U*, the genes employ 0 to 7, where 0 represents excluding rule and others indicate adopting rules. Then the rules in Table 1 can be encoded as 0020100, 0300000, 0000000, 0005000, 0000400, 0000070, and 0000006. *H* and *Y* all have 7 linguistic values, so the length of a chromosome is 49.

#### 3.2.2. Encoding of Membership Function

If the parameters of membership functions are encoded directly for tuning, there would be too many constraints which may require extremely large searching space and deteriorate the learning performance. The encoding method proposed by Chiou and Lan [18] can overcome the problem of deteriorating caused by incorporating all the constraints. Let parameters *x*_*k*_^*l*^, *x*_*k*_^*m*^, and *x*_*k*_^*r*^ represent left anchor, middle anchor, and right anchor of *k*th linguistic values, respectively. They should satisfy the following relations:

(1) xkl≥xkm≥xkr,xkl≥xk−1l,xkm≥xk−1m,xkr≥xk−1r.

**Figure 6:**
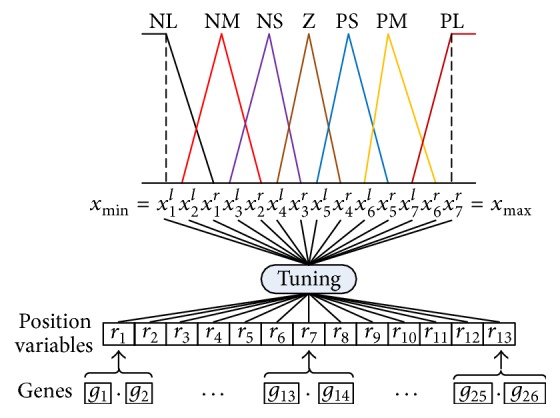
The encoding method for membership function.

The encoding method is depicted in Figure 6 and the constraints can be listed as follows:

(2) xmaxx7m=x7r≥x6r≥x7lx5r≥x6lx4r≥x5lx3r≥x4lx2r≥x3lx1r≥x2l≥x1m=x1l=xmin,xkmxkr+xkl2,k=2,3,4,5,6.

The orders between *x*_5_^*r*^ and *x*_7_^*l*^, *x*_4_^*r*^ and *x*_6_^*l*^, *x*_3_^*r*^ and *x*_5_^*l*^, *x*_2_^*r*^ and *x*_4_^*l*^, and *x*_1_^*r*^ and *x*_3_^*l*^ are indeterminate. To tune these parameters, position variables *r*_1_ ~ *r*_13_ are designed as follows:

(3) x2l=xmin+r1×θ,x1r=x2l+r2×θ,x3l=x2l+r3×θ,xir=max⁡xi−1r,xi+1l+r2i×θ,i=2,3,4,5,6,xnl=max⁡xn−3r,xn−1l+r2n−3×θ,n=4,5,6,7,θ=kxmax−xmin∑i=113ri,

where contraction factor *k* is proposed to avoid the fuzzy field going out of range [*x*_min_, *x*_max_]. After balancing the memory consumption and the accuracy, we decide every position variable range from 0.0 to 9.9, each position variable is represented by 2 genes, one gene is for the unit, another is for the first decimal, and each gene ranges from 0 to 9. Thus, 26 genes are needed to encode the membership functions of one variable and a chromosome is composed of 78 genes for 3 variables.

### 3.3. Constituents of Genetic Algorithm

#### 3.3.1. Selection Operation

There are several methods for selection: roulette wheel selection (RWS) method, tournament method, and ranking selection, The RWS method is most commonly used. In order to select the high-fitness individual, a tangent function is used in the RWS, named tangent function RWS (Tan-RWS). The selection procedure of Tan-RWS is described as follows.

**Step 1 (calculate the fitness of chromosomes in initial population).**

The GFLC model aims to minimize the mean absolute deviation of coal floor height between implementation stage and demonstration stage. The objective function of the *n*th chromosome can be given as follows:

(4) $$\begin{array}{c} f_{n}=\frac{\sum_{i=1}^{j}\left|C_{i,j}+U_{n,j}-C_{1,j}\right|}{j}\;n=1,2,\ldots ,P_{size}, \end{array}$$

where *j* is the sampling points, *C*_*i*,*j*_ represents the coal floor height in stage *i*, *U*_*n*,*j*_ represents the adjustment of coal floor height in next implementation stage, and *P*_size_ is the population size. The fitness function of the *n*th chromosome can be calculated as follows:

(5) $$\begin{array}{c} {fit}_{n}=\frac{1}{f_{n}}\;n=1,2,\ldots ,P_{size}. \end{array}$$

**Step 2 .**

Replace fit_*n*_ by *P*_*n*_ as follows:

(6) Pn=tan⁡π2×fitn−fminfmax−fminfmin≤fitn<fmax1fitn=fmaxn=1,2,…,Psize.

**Step 3 .**

Calculate the cumulative value as follows:

(7) $$\begin{array}{c} C=\overset{P_{size}}{\underset{n=1}{\sum }}P_{n}. \end{array}$$

**Step 4 .**

Generate a random number *r* in the range [0,1] for *P*_size_ times. Divide [0,1] into *P*_size_ parts according to the percentage of *P*_*n*_ in *C*; then each chromosome has particular interval. If *r* ∈ [∑_*k*=1_^*n*−1^ *P*_*k*_/*C*, ∑_*k*=1_^*n*^ *P*_*k*_/*C*), then *n*th individual is selected.

Obviously, the tangent function of low-fitness chromosome is smaller than the high-fitness one; thus the high-fitness chromosome is easy to be picked out.

#### 3.3.2. Crossover Operation

Uniform crossover is proved to be the most powerful crossover allowing the offspring chromosomes to search all possibilities of recombining those different genes in parents [30–33]. To improve the possibilities of generating the best individuals, uniform crossover with comparison is adopted in this paper. The comparison strategy is carried out before uniform crossover, which can be given as follows.

*G*_*w*_^*t*^ = {*g*_*w*1_^*t*^,…, *g*_*wk*_^*t*^,…, *g*_*wK*_^*t*^} and *G*_*v*_^*t*^ = {*g*_*v*1_^*t*^,…, *g*_*vk*_^*t*^,…, *g*_*vK*_^*t*^} are two parents for crossover and *t* is the generation number. *G*_*w*_^*t*+1^ = {*g*_*w*1_^*t*+1^,…, *g*_*wk*_^*t*+1^,…, *g*_*wK*_^*t*+1^} and *G*_*v*_^*t*+1^ = {*g*_*v*1_^*t*+1^,…, *g*_*vk*_^*t*+!^,…, *g*_*vK*_^*t*+1^} are two offspring.

The following rules can be generated according to the comprising results of the alleles for two parents.

 If |*g*_*wk*_^*t*^ − *g*_*vk*_^*t*^| < 2, then *g*_*wk*_^*t*+1^ = *g*_*vk*_^*t*^, *g*_*vk*_^*t*+1^ = *g*_*wk*_^*t*^.
 If |*g*_*wk*_^*t*^ − *g*_*vk*_^*t*^| ≥ 2 and *b*_*k*_ = 1, then *g*_*wk*_^*t*+1^ = *g*_*vk*_^*t*^, *g*_*vk*_^*t*+1^ = *g*_*wk*_^*t*^.
 If |*g*_*wk*_^*t*^ − *g*_*vk*_^*t*^| ≥ 2 and *b*_*k*_ = 0, then *g*_*wk*_^*t*+1^ = *g*_*wk*_^*t*^, *g*_*vk*_^*t*+1^ = *g*_*vk*_^*t*^.

|*g*_*wk*_^*t*^ − *g*_*vk*_^*t*^| reflects the similarity of these two alleles; the small value of |*g*_*wk*_^*t*^ − *g*_*vk*_^*t*^| means much similarity of these two alleles. To ensure the sufficient search space of GA, the similar alleles need to be crossed. *b*_*k*_ randomly takes a binary of 0 or 1.

#### 3.3.3. Mutation Operation

Mutation is a method of generating new chromosomes to explore new regions of the search space [34]. Nonuniform mutation was developed by Michalewicz [35] to tackle numerical parameter optimization problems. This operator is a dynamical and adaptive mutation operator which can decrease the disadvantage of random mutation in GA. However, the encoding method of fuzzy logic rules and membership function in this paper is based on integer, so the value of gene selected for mutation should be rounded to the nearest integer. It can be depicted as follows.

*G*_*w*_^*t*^ = {*g*_*w*1_^*t*^,…, *g*_*wk*_^*t*^,…, *g*_*wK*_^*t*^} is a chromosome and the gene *g*_*wk*_^*t*^ is selected for mutation (the domain of *g*_*wk*_^*t*^ is [*g*_*wk*_^*l*^, *g*_*wk*_^*u*^]); the value of *g*_*wk*_^*t*+1^ can be calculated as follows:

(8) $$\begin{array}{c} g_{wk}^{t+1}=\left\{\begin{array}{cc} \left[g_{wk}^{t}+\Delta \left(t,g_{wk}^{u}-g_{wk}^{t}\right)+0.5\right] & \text{if }b=0,\\ \left[g_{wk}^{t}-\Delta \left(t,g_{wk}^{t}-g_{wk}^{l}\right)+0.5\right] & \text{if }b=1, \end{array}\right. \end{array}$$

where *b* randomly takes a binary value of 0 or 1. The function Δ(*t*, *z*) returns a value in the domain of [0, *z*], so the probability of Δ(*t*, *z*) approaches to 0 as *t* increases:

(9) $$\begin{array}{c} \Delta \left(\;t,z\;\right)=z\left(\;1-r^{{\left(1-t/T\right)}^{h}}\;\right), \end{array}$$

where *r* randomly takes a real number in the range of [0,1]; *T* is the maximum number of generations; and *h* is a system parameter determining the degree of dependency on the iteration number. To get good nonuniform mutation performance, the value of *h* should be set in the range of [0.5,1].

#### 3.3.4. The Adaptive Probability of Crossover and Mutation

According to [23, 36], the adaptive probability of crossover and mutation can not only maintain diversity in the population but also sustain the convergence capacity of GA. Thus this adaptive improvement is adopted in this paper, the crossover probability *P*_*c*_, and the mutation probability *P*_*m*_ can be expressed as follows:

(10) Pc=Pc1−Pc1−Pc2fmax−f′fmax−favg,f′≥favgPc1,f′<favgPm=Pm1−Pm1−Pm2fmax−ffmax−favg,f≥favgPm1,f<favg.

According to [23], *P*_*c*1_ = 0.9, *P*_*c*2_ = 0.6, *P*_*m*1_ = 0.1, *P*_*m*2_ = 0.001, *f*_max_ is the highest value of fitness in the population, *f*_avg_ represents average value of fitness in the population, *f*′ denotes higher fitness one in two crossover individuals, and *f* is the fitness value of mutation individual.

#### 3.3.5. The Iterative Evolution Algorithm

**Figure 7:**
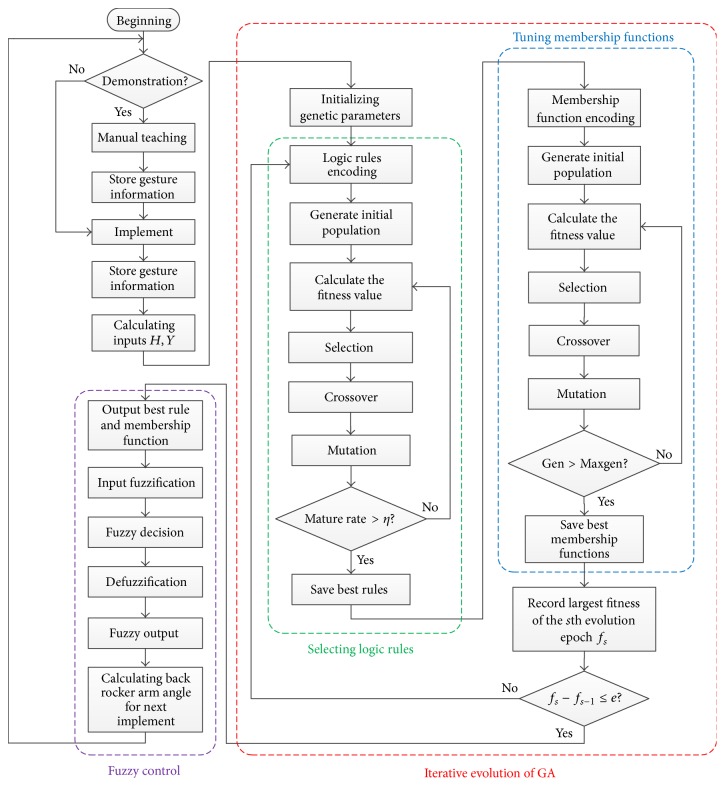
Flowchart of the proposed approach.

If both components are learned simultaneously, a very long chromosome is often needed; thus it could deteriorate the learning performance. The bilevel iterative evolution proposed by Chiou and Lan can learn logic rules and membership functions sequentially without subjectively presetting both of them [18]. The stop condition is set based on maximum number of generations and the iterative evolution procedure can be depicted in Figure 7.

In Figure 7, Maxgen, Gen, *f*_*s*_, *η*, and *e* denote the maximum number of generations, the number of generations, the largest fitness of the *s*th evolution epoch, the maximum mature rate, and a number less than 1, respectively.

### 3.4. Flowchart of the Proposed Approach

According to the above description of proposed approach based on genetic fuzzy logic control, the flowchart of the proposed approach can be summarized in Figure 7.

## 4. Simulations and Discussion

### 4.1. Justification of Tan-RWS

**Figure 8:**
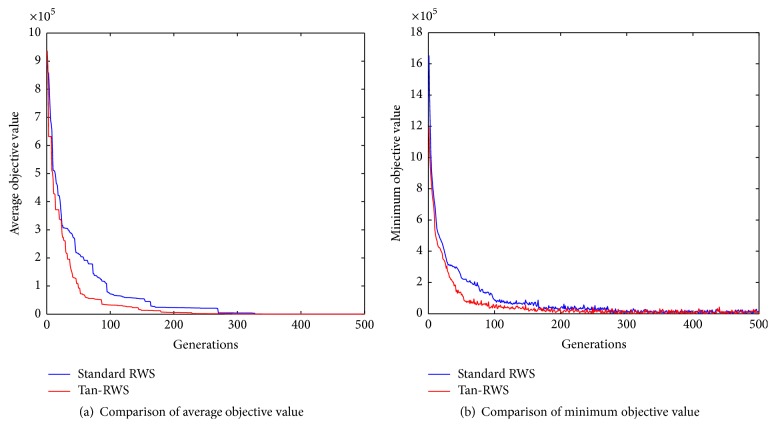
Justifications of Tan-RWS.

To justify the performance of Tan-RWS, an example is given. De Jong function can be expressed as follows:

(11) $$\begin{array}{c} f\left(\;x\;\right)=\overset{n}{\underset{i=1}{\sum }}x_{i}^{2},\;-512\leq x_{i}\leq 512, \end{array}$$

where *n* is the dimension, we set *n* = 20 and look for min⁡*f*(*x*), and the theoretical minimum is *f*(0,0,…, 0) = 0. Single point crossover and basic bit mutation are employed; each individual is encoded by binary and the encoding precious is 20. Other parameters are set as follows: number of individuals NIND = 40, maximum number of generations Maxgen = 500, probability of crossover *P*_*c*_ = 0.7, and probability of mutation *P*_*m*_ = 0.035. Standard RWS and Tan-RWS are, respectively, taken as the select operator, and the results are shown in Figure 8. From Figure 8, it can be obtained that the GA with Tan-RWS has faster convergence rate than the GA with standard RWS and they all get the global solution.

### 4.2. Simulation of the Proposed Approach

#### 4.2.1. Preparation

**Figure 9:**
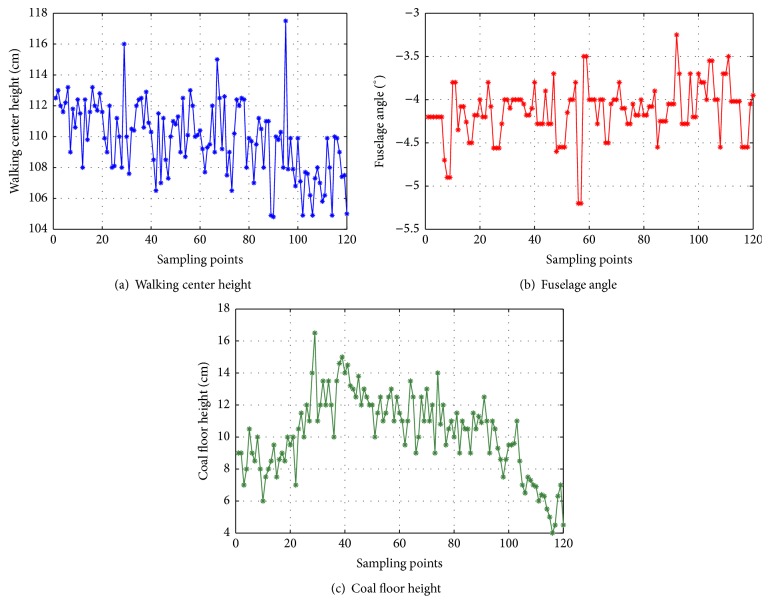
Gesture information in demonstration stage.

The sample data are measured in a real shearer. The sample data can be collected by the sensors fixed in the shearer, and these data are saved in the storage unit of shearer controller (S7-300 PLC); then the data are sent to the upper computer via the industrial Ethernet. The sample data of the walking center height, the fuselage angle, and the coal floor height can be shown in Figure 9.

**Figure 10:**
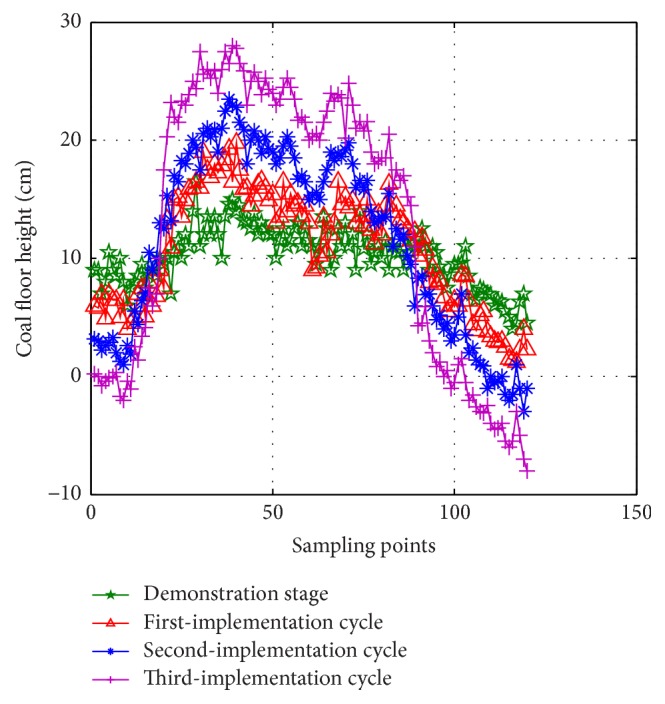
Coal floor height of traditional memory cutting.

According to the gesture information in demonstration stage, the shearer works along the coal wall in traditional memory cutting method automatically for 3 implementation cycles, as shown in Figure 10. The compared data of coal floor height are listed in Table 2. Seen from Figure 10 and Table 2, the enlargement of deformation gets larger and the standard deviation increases from 2.48 cm to 11.77 cm.

#### 4.2.2. Simulation Results

**Figure 11:**
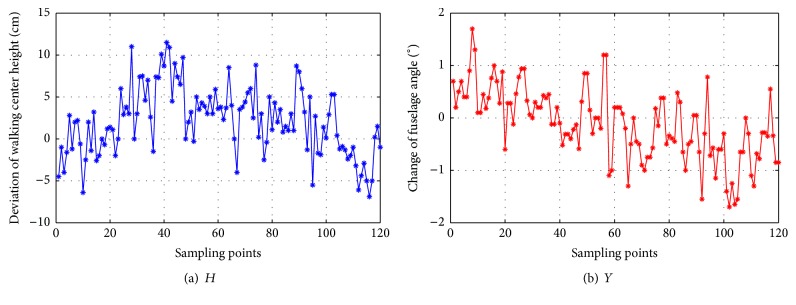
Inputs of GFLC.

The walking center height and the fuselage angle in first-implementation cycle can be calculated according to the geometric parameters of shearer and the coal floor height in demonstration stage. Then, *H* and *Y* can be calculated and taken as the inputs of GFLC, as shown in Figure 11.

**Figure 12:**
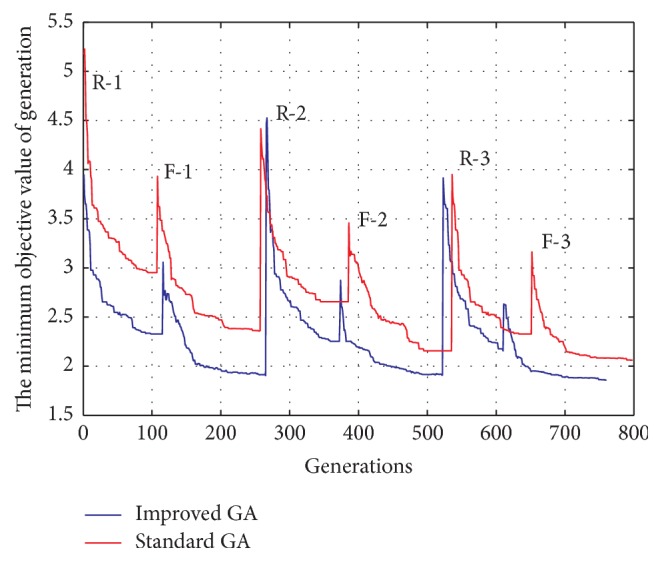
The comparison of iterative process. R-# represents the #th evolution of selecting logic rules. F-# represents the #th evolution of tuning membership functions.

**Figure 13:**
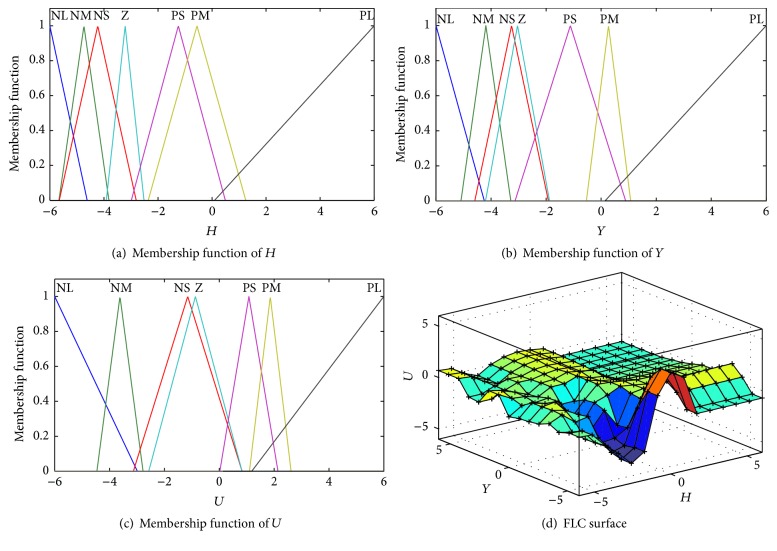
Membership functions and surface tuned by improved GA.

To guarantee the operation speed and performance of the algorithm, we take repeated experiments and determine the parameter values generally: *n* = 30, mature rate *η* = 70%, Maxgen = 150, *e* = 0.05, *h* = 0.5, and contraction factor *k* = 0.67.  Figure 12 describes the comparison of iterative process, R-# represents the #th evolution of selecting logic rules, and F-# represents the #th evolution of tuning membership functions. From Figure 12, improved GA converges after three iterative evolutions with 760 generations, standard GA converges with 798 generations, the improved GA has faster converge rate, and the solution of improved GA is better than standard GA. The reason of objective function jumping is the sequential optimization. The shapes of membership functions and the FLC surface tuned by improved GA are plotted in Figure 13.

**Figure 14:**
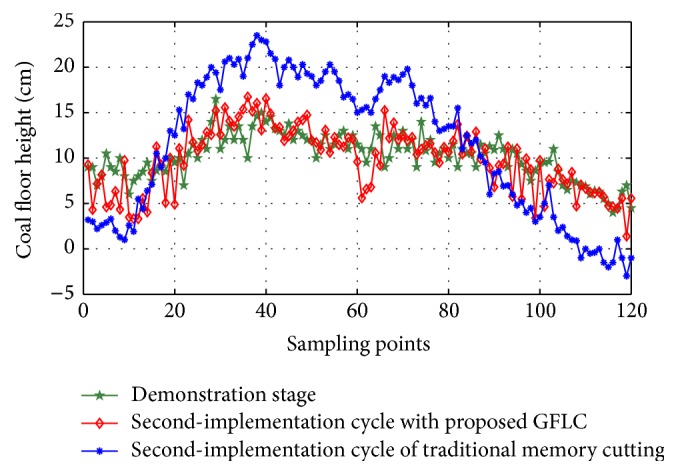
Simulation results of proposed approach in second-implementation cycle.

Then, the coal floor height in second-implementation cycle can be optimized and simulated by the use of GFLC. The simulation results are shown in Figure 14. Our aim is to correct the floor shape in implementation cycles similar to the floor shape in demonstration cycle. Seen from Figure 14, the floor shape of second-implementation cycle with proposed GFLC is closer to the floor shape of demonstration stage, better than traditional memory cutting method.

**Figure 15:**
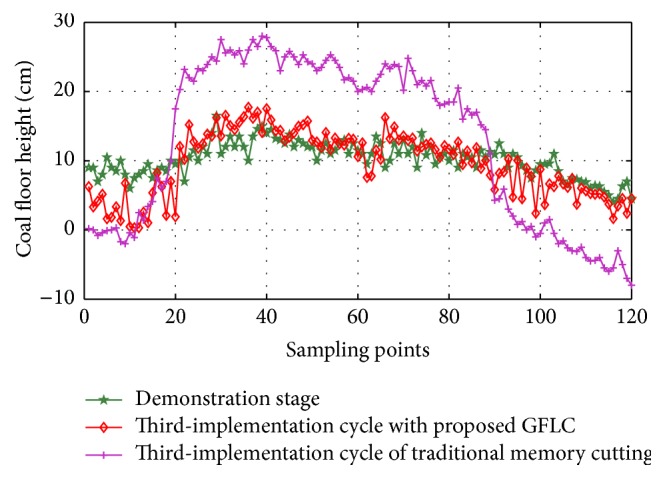
Simulation results of proposed approach in third-implementation cycle.

Repeating the proposed approach, the third-implementation cycle simulation results are given in Figure 15; the performance is also good.

#### 4.2.3. Comparison with Traditional Fuzzy Logic Control

**Figure 16:**
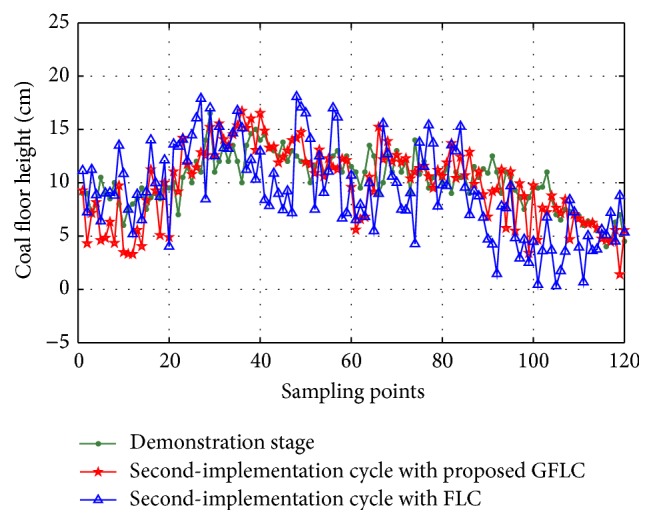
Comparison of simulation results in second-implementation cycle.

**Figure 17:**
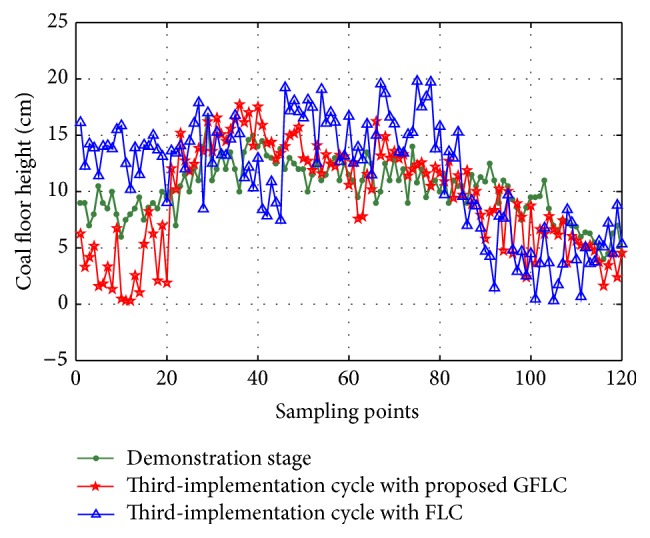
Comparison of simulation results in third-implementation cycle.

In order to demonstrate the effectiveness of proposed method, the traditional fuzzy logic control (FLC) method and proposed method are provided to solve the problem of the above example. The configurations of simulation environment for two algorithms are uniform and the relevant parameters are in common with the above example. The compared results are shown in Figures 16 and 17 and Table 3. It can be observed that the standard deviation of GFLC is smaller than that of FLC. Thus, GFLC is a more efficient approach than FLC to reduce the enlargement of coal floor deformation in shearer memory cutting process.

## 5. Industrial Application

**Figure 18:**
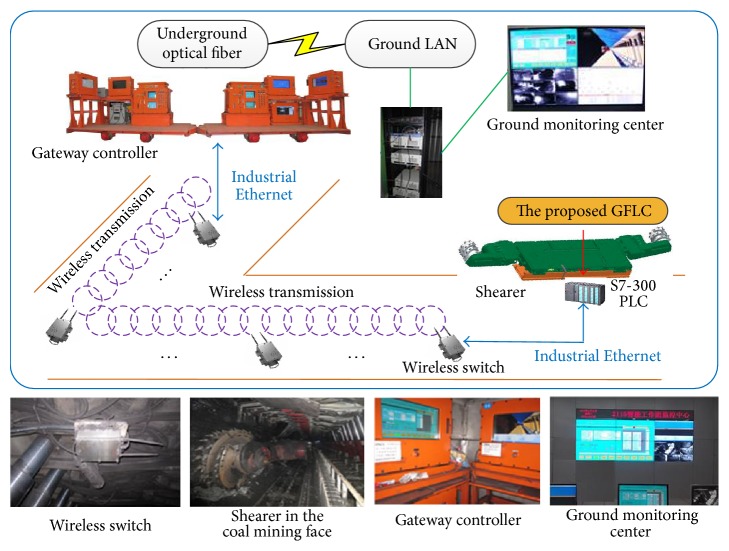
Industrial application example of proposed method.

In this section, a system based on proposed approach has been developed and applied in the field of coal mining face as shown in Figure 18.

As shown in Figure 18, the proposed approach is uploaded into the S7-300 PLC (Programmable Logic Controller). The real time operating data can be measured by the multiple sensors fixed in the shearer, and then these data are transferred from PLC to the “gateway controller” through the industrial Ethernet and wireless network; the “Ground monitoring center” receives these data through the communication of the underground optical fiber and the ground LAN. The “gateway controller” and “ground monitoring center” are used to control and monitor the shearer working state.

**Figure 19:**
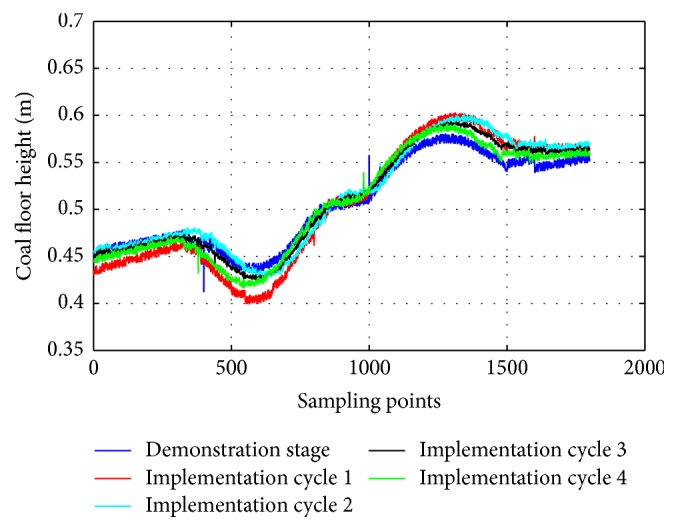
Application effect of proposed system.

For the shearer, the aim is to correct the floor shape in implementation cycles similar to the floor shape in demonstration cycle. In order to illustrate the application effect of proposed system, the coal floor height is collected by 1 Hz, 30 minutes is needed to accomplish one cycle, and the collected data are transmitted to the “gateway controller” and “ground monitoring center.” The change curves of coal floor height in 4 sequential implementation cycles are plotted to illustrate the application effect of proposed system, as shown in Figure 19.

Seen from Figure 19, the deformations exist in the demonstration stage, and the deformations are enlarged in implementation cycle 1. The proposed approach can influence the control effect of coal floor shape from implementation cycle 2, and the coal floor shape gets more similar to the coal floor shape of demonstration stage with implementing more implementation cycles. The application effect indicates that the system based on proposed method can deal with the coal floor deformation and its enlargement.

## 6. Conclusions and Future Work

In order to reduce the enlargement of coal floor deformation in shearer memory cutting process, this paper proposed a control approach based on improved genetic algorithm and fuzzy logic control (GFLC). The framework of proposed approach is presented. Several strategies such as uniform crossover, nonuniform mutation, and adaptive probability of crossover and mutation are employed and the Tan-RWS is proposed to improve GA. Improved GA is used to optimize the logic rules and membership functions of FLC subsequently. To demonstrate the performance of proposed method, two simulation examples are provided and the comparison with common FLC is carried out. Finally, an industrial application example of coal mining face is demonstrated to specify the effect of proposed system. The results verify that the GFLC method is an effective support tool for decreasing the enlargement of coal floor deformation in shearer memory cutting process.

In future studies, the constituents of GA should be studied with further research and the improvements for GA with best performance should be analyzed. The solution for coal floor deformation and its enlargement in this paper may not be perfect, so we need to evaluate other optimization algorithms to enhance FLC, including particle swarm optimization algorithm, ant colony algorithm, and artificial fish swarm algorithm. Other control methods are also needed to be studied, such as self-adaptation control and predictive control.

## Figures and Tables

**Table 1:** Fuzzy control table.

*U*		*H*						
		NL	NM	NS	Z	PS	PM	PL
*Y*	NL	0	0	2 (NM)	0	1 (NL)	0	0
	NM	0	3 (NS)	0	0	0	0	0
	NS	0	0	0	0	0	0	0
	Z	0	0	0	5 (PS)	0	0	0
	PS	0	0	0	0	4 (Z)	0	0
	PM	0	0	0	0	0	7 (PL)	0
	PL	0	0	0	0	0	0	6 (PM)

**Table 2:** Compared data of traditional memory cutting.

Items	Average value (cm)	Standard deviation (cm)
Demonstration stage	10.19	2.48
First-implementation cycle	10.86	4.96
Second-implementation cycle	11.48	7.76
Third-implementation cycle	12.94	11.77

**Table 3:** The compared results of GFLC and FLC.

Items	Average value (cm)	Standard deviation (cm)
Demonstration stage	10.19	2.48
Second-implementation cycle with proposed GFLC	9.81	3.55
Second-implementation cycle with FLC	9.27	4.21
Third-implementation cycle with proposed GFLC	9.54	4.68
Third-implementation cycle with FLC	11.54	5.03
